# Lymphoid Tissue Damage in HIV-1 Infection Depletes Naïve T Cells and Limits T Cell Reconstitution after Antiretroviral Therapy

**DOI:** 10.1371/journal.ppat.1002437

**Published:** 2012-01-05

**Authors:** Ming Zeng, Peter J. Southern, Cavan S. Reilly, Greg J. Beilman, Jeffrey G. Chipman, Timothy W. Schacker, Ashley T. Haase

**Affiliations:** 1 Department of Microbiology, Medical School, University of Minnesota, Minneapolis, Minnesota, United States of America; 2 Division of Biostatistics, School of Public Health, University of Minnesota, Minneapolis, Minnesota, United States of America; 3 Department of Surgery, Medical School, University of Minnesota, Minneapolis, Minnesota, United States of America; 4 Department of Medicine, Medical School, University of Minnesota, Minneapolis, Minnesota, United States of America; Emory University, United States of America

## Abstract

Highly active antiretroviral therapy (HAART) can suppress HIV-1 replication and normalize the chronic immune activation associated with infection, but restoration of naïve CD4^+^ T cell populations is slow and usually incomplete for reasons that have yet to be determined. We tested the hypothesis that damage to the lymphoid tissue (LT) fibroblastic reticular cell (FRC) network contributes to naïve T cell loss in HIV-1 infection by restricting access to critical factors required for T cell survival. We show that collagen deposition and progressive loss of the FRC network in LTs prior to treatment restrict both access to and a major source of the survival factor interleukin-7 (IL-7). As a consequence, apoptosis within naïve T cell populations increases significantly, resulting in progressive depletion of both naïve CD4^+^ and CD8^+^ T cell populations. We further show that the extent of loss of the FRC network and collagen deposition predict the extent of restoration of the naïve T cell population after 6 month of HAART, and that restoration of FRC networks correlates with the stage of disease at which the therapy is initiated. Because restoration of the FRC network and reconstitution of naïve T cell populations are only optimal when therapy is initiated in the early/acute stage of infection, our findings strongly suggest that HAART should be initiated as soon as possible. Moreover, our findings also point to the potential use of adjunctive anti-fibrotic therapies to avert or moderate the pathological consequences of LT fibrosis, thereby improving immune reconstitution.

## Author Summary

The hallmark of HIV-1 infection is depletion of CD4 T cells, whose loss leads to the opportunistic infections and cancers characteristic of AIDS. Highly active antiretroviral therapy (HAART) can control HIV-1 replication, but reconstitution particularly of naïve T cells is often incomplete and slow. We show here that fibrosis damages lymphoid tissues (LT), thereby contributing to depletion and incomplete reconstitution. Prior to treatment, chronic immune activation induces LT fibrosis to disrupt the fibroblastic reticular cell (FRC) network, the major source of the T cell survival factor interleukin 7 (IL-7). Fibrosis in this way interferes with the access of T cells to IL-7 “posted” on the FRC network. Without a source and access to IL-7, naïve cells are depleted prior to initiating HAART because of increased apoptosis, and, even after initiating HAART, the losses continue by this mechanism because of pre-existing LT damage. Thus, LT fibrosis impairs immune reconstitution despite the beneficial effects of HAART in suppressing viral replication. Because less LT damage has accumulated in earlier stages of infection, early initiation of HAART also improves immune reconstitution. This LT damage mechanism also suggests that anti-fibrotic treatment in addition to HAART could further improve immune reconstitution.

## Introduction

The hallmark of HIV-1 infection, depletion of CD4^+^ T cells, has been largely attributed to direct mechanisms of infection and cell death from viral replication or killing by virus-specific cytotoxic T-lymphocytes (CTLs), and to indirect mechanisms such as increased apoptosis accompanying chronic immune activation associated with HIV-1 infections [1]. It is thus puzzling that if these were the sole mechanisms responsible for CD4^+^ T cell depletion, why 20% of HIV-1 infected patients have no significant increase in their peripheral blood CD4 count after initiation of HAART, since treatment can suppress viral replication to undetectable levels and normalize much of the chronic immune activation associated with infection [2]–[3]. Moreover, even among patients with significant increases in peripheral blood CD4^+^ T cells, few reconstitute to normal levels after years of HAART, and this incomplete immune reconstitution is associated with significantly higher rates of malignancy and other morbidities compared with HIV-uninfected individuals [4]–[11].

The preferential depletion of naïve T cells in blood and lymphoid tissues (LT) [3], where they mainly reside, also poses particular difficulties for attributing depletion simply to direct mechanisms of viral infection or indirect mechanisms of activation-induced cell death (AICD), since (1) naïve CD4^+^ T cells are resistant to HIV-1 infection and (2) AICD should primarily affect the activated effector and memory populations [12]–[14]. Furthermore, the similar extent of depletion of not only naïve CD4^+^ T cells but also naïve CD8^+^ T cells that are not usually infected by HIV [15]–[16], suggests that there is a general mechanism impacting naïve T cell populations unrelated to direct infection.

The incomplete restoration of naïve T cell populations with HAART also points to mechanisms in addition to AICD in depletion of CD4^+^ T cells, since suppression of this “drain” should enable repopulation of naïve CD4^+^ T cell populations by thymopoiesis and homeostatic proliferation of existing naïve T cells in secondary LT, but this does not happen many patients [17]–[20]. Cumulative observations therefore suggest that there may be additional mechanisms that impair the survival of naïve T cells, thereby restricting immune reconstitution [1], [3].

To account for the preferential loss of naïve T cells, and failure of HAART to restore both naïve and memory populations by thymopoesis and homeostatic proliferation, we have proposed a damaged LT niche hypothesis in which collagen deposition disrupts the FRC network on which naïve T cells migrate and gain access to survival factors such as interleukin-7 (IL-7). This results in elevated levels of naïve T cell apoptosis [21]–[23] before treatment and impairs the reconstitution of naïve T cells after treatment. We recently showed in the SIV-rhesus macaque animal model that the critical disruption in LT architecture caused by collagen deposition and decreased access of T cells to survival factor IL-7 “posted” on the FRC network was in fact associated with increased apoptosis and depletion of both naïve CD4^+^ and CD8^+^ T cells. We also showed that this mechanism is a cooperative and cumulative vicious cycle in which the mutual interdependencies for survival of naïve T cells on IL-7, and the FRC network on lymphotoxin-beta (LT-β) supplied by the T cells, perpetuate depletion of both T cells and the FRC network [24].

One implication of this model is that because the impact of LT fibrosis on CD4^+^ T cell depletion is progressive and cumulative, initiating HAART in the early stages of infection should improve immune reconstitution because there should be less collagen deposition and loss of the FRC network at this stage. We tested this hypothesis by examining LTs from HIV-1 infected individuals at baseline and 6 months after initiating HAART in the acute, pre-symptomatic and AIDS stages of infection. We first document the same damaged LT niche mechanism described in the SIV-rhesus macaque model of collagen deposition and loss of the FRC network with depletion of naïve T cells through increased apoptosis as a consequence of decreased access to IL-7. We then show that the extent of loss of the FRC network and collagen deposition predict the extent of inhibition of naïve T cell apoptosis and restoration of the naïve T cell population in LT after 6 months of HAART, and total CD4 T cell counts in peripheral blood after 12 months of HAART. Furthermore, we find that the extent of restoration of FRC network after 6 months of HAART is highly dependent on the stage of disease at which the therapy is initiated, with greatest restoration only when HAART is initiated during the early stage of infection. This directly correlates with optimal inhibition of naïve T cell apoptosis and restoration of naïve T cells in the patients receiving HAART during the early stage of infection. This mechanism explains why initiation of HAART during the early stage of infection is associated with more rapid and complete CD4^+^ T cell restoration, and thus strongly argues for early initiation of HAART [25]–[26]. It also argues for a potential use of adjunctive therapies such as anti-fibrotic therapy to avert and/or revert the LT structure to improve immune reconstitution.

## Results

### Collagen deposition and loss of the FRC network impede access to and source of IL-7 in HIV-1 infection

**Figure 1:**
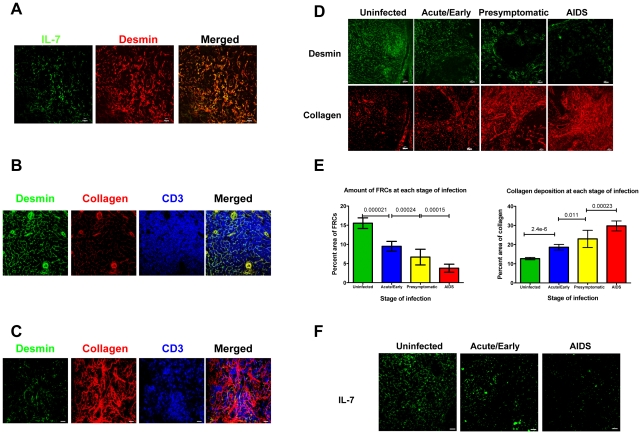
Collagen deposition and loss of the FRC network impede access to and source of IL-7 in HIV-1 infection. A. FRCs are the major producers of IL-7. LN sections (representative image for one HIV negative subject of 5) stained for desmin (red) and IL-7 (green). Merged confocal image shows co-localization of IL-7 and FRCs in T cell area. Scale bar, 10 µm. B–C. Collagen deposition and loss of the FRC network disrupts interaction between T cells and FRCs. Confocal images of LN sections from an uninfected subject (representative image for one subject of 5) immunofluorescently stained for desmin (green), collagen (red) and CD3 (blue). The merged image shows that FRCs colocalize with collagen and T cells are in contact with the FRC network (B). Confocal images of LN sections from a subject at AIDS stage (representative image for one subject of 6). The merged image shows that the loss of FRCs and associated collagen deposition leads to loss of the contact between FRCs and T cells, which instead contact mainly extra-FRC collagen. Scale bar, 20 µm (C). D. Confocal images of LN sections from HIV negative subjects and from subjects at different stage of HIV infection immunofluorescently stained for desmin (green) and collagen (red), showing the gradual loss of FRCs in the T cell zone within LTs, which is associated with extensive collagen deposition during HIV infection. Scale bar, 20 µm. E. Quantification of average amount of FRCs and collagen deposition at each stage of infection, showing the gradual loss of FRCs and collagen deposition. Error bars represent the s.d. F. Confocal images of LN sections from HIV negative subjects and from subjects at different stage of HIV infection immunofluorescently stained for IL-7 (green), showing that the gradual loss of IL-7 in the T cell zone is associated with gradual depletion of FRCs (E). Scale bar, 20 µm.

To evaluate this hypothesis, we first show that the FRC network is the major source of IL-7 for T cells in human LTs, as has been demonstrated in mice and monkeys [22], [24]. In LT sections from uninfected individuals stained for IL-7 and desmin, a marker for FRCs, IL-7 largely co-localizes with the FRC network on which lymphocytes, antigen presenting cells and other cells within LTs migrate (Figure 1A). This architecture allows T cells to efficiently access survival factors such as IL-7 and self-antigen-major histocompatibility complex as well as chemokines “posted” on their path. Thus, in the lymph node (LN) sections from HIV-1-uninfected individuals stained for type I collagen, desmin and T cells, the collagen within the FRC network co-localizes with desmin, and the T cells visibly contact the FRC network (Figure 1B). In contrast, HIV-1 infection is associated with stage-specific progressive decreases in the FRC network (Figure 1D-E) and thus the available source of IL-7 (Figure 1F). The depletion of FRCs correlates with a parallel increase in collagen deposited outside the FRC network, so that as infection progresses, fewer and fewer T cells are in contact with and have access to IL-7 on the FRC network (Figure 1C) compared with uninfected populations (Figure 1B).

### T cell apoptosis increases with decreased availability of IL-7

**Figure 2:**
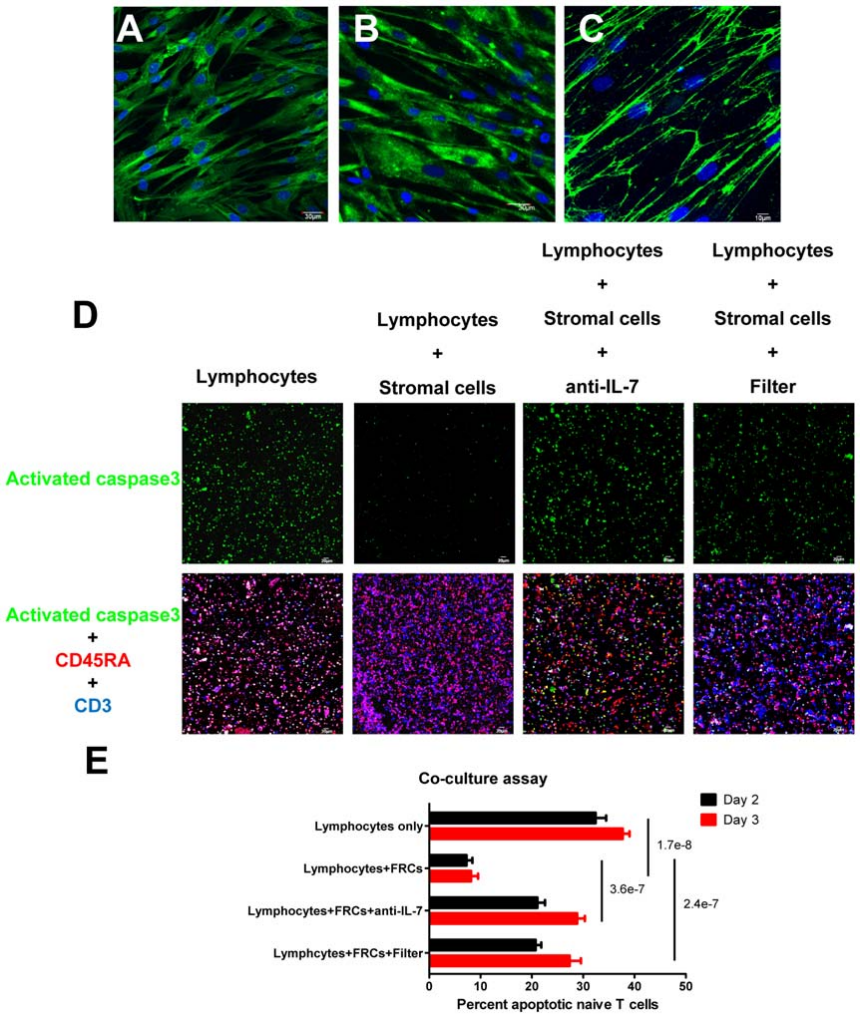
Naïve T cells need to contact FRCs to get access to IL-7 for survival. (A–C) IL-7 is produced and presented on the surface of stromal cells. A-B. Confocal images of monolayer of fixed and permeablized stromal cells isolated from human tonsil immunofluorescently stained for (A) IL-7 (green) or (B) desmin (green) and DAPI (blue) at one-day post passage. Scale bar, 30 µm. C. Confocal image of live stromal cells (DAPI: blue) staining showing the IL-7 (green) on the surface of stromal cells. Scale bar, 10 µm. D-E. FRC-like stromal cells enhance the survival of naïve T cells via IL-7. D. Triple fluorescently stained activated caspase 3^+^ (green), CD45RA^+^ (red) and CD3^+^ (blue) cells in an ex vivo culture system showing that stromal cells enhance the survival of naïve T cell by mechanisms dependent on IL-7 and cell contact. 2x10^5^ lymphocytes from human tonsil were cultured with or without stromal cells for 2–3 days. Naïve T cell apoptosis is reduced in co-cultures with stromal cells (+ stromal cells) compared to cultures without stromal cells. Apoptosis in the naïve T cell population increases with IL-7 blocking antibody (anti-IL-7) or when lymphocytes are separated from stromal cells by a transwell filter (Filter) compared to co-cultures with stromal cells. Scale bar, 60 µm. E. Quantification of the percentages of activated caspase 3^+^CD45RA^+^CD3^+^ naïve T cells in total T cell population at day 2 and day 3 cultures. Values are the mean of the percent apoptotic naïve T cells ± s.d. ANOVA comparison was done on the average percentages of day 2 and day 3.

Because survival of naïve T cells is dependent on access to IL-7 [21]–[23], the collagen deposition-restricted access to and loss of the FRC network itself should result in an increase in apoptosis proportional to the extent of collagen deposition and decreased IL-7 source as infection progresses. We first demonstrate that contact with FRCs as a source of IL-7 is critical for T cell survival in an ex vivo culture system. We established monolayers of desmin^+^ IL-7^+^ FRC-like cells from stromal cells isolated from human tonsils (Figure 2A–B), and show that IL-7 localizes to the surfaces of live cells stained without permeabilization (Figure 2C). Only about 10% of naïve T cells underwent apoptosis if co-cultured with autologous IL-7^+^ FRC-like cells compared to about 30–40% of naïve T cells cultured for 2–3 days without stromal cells. We show that the enhanced survival is contact dependent and is mediated mainly via IL-7, as antibody blocking of IL-7 or separation of T cells and the FRCs by transwells leads to increased apoptosis in naïve T cells (Figure 2D–E). However, the blockade of IL-7 does not fully recapitulate the apoptosis level in the naïve T alone culture, suggesting that other survival factors such as CCL19 produced by FRCs may independently support the survival of naïve T cells [22].

**Figure 3:**
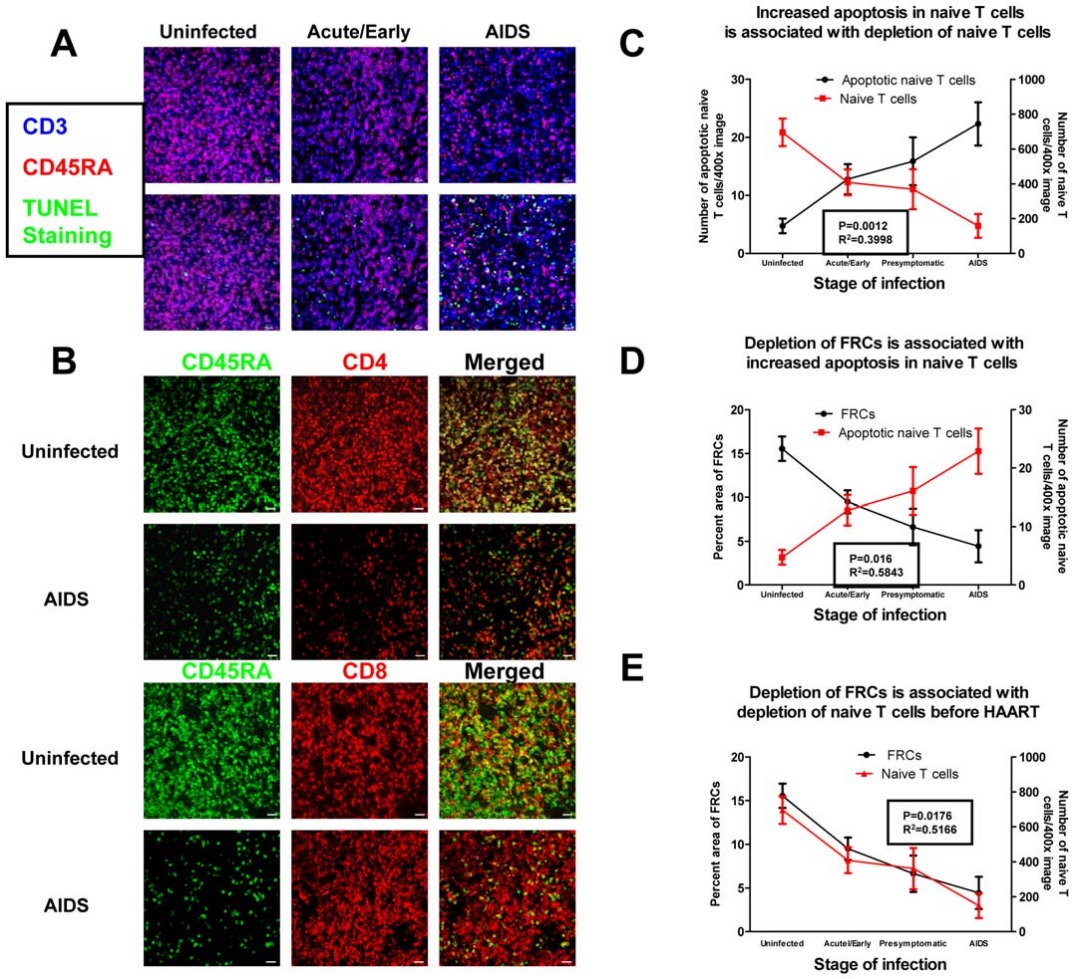
Loss of FRCs is associated with loss of naïve T cells within LTs. A. Confocal images of LN sections from subjects at different stage of HIV infection triple immunofluorescently stained for TUNEL (green), CD45RA (red) and CD3 (Blue), showing the gradual loss of CD45RA^+^CD3^+^ naïve T cells is associated increased apoptosis in the naïve T cell population within LTs during HIV infection. Scale bar, 10 µm. B. Confocal images of LN sections from subjects at different time points post HIV infection double immunofluorescently stained for CD45RA (green) and CD4 or CD8 (red), showing both naïve CD4 and CD8 T cells are depleted within LTs. Scale bar, 20 µm. C. Quantitative image analysis of the number of apoptotic naïve T cells and the number of naïve T cells (CD45RA^+^CD3^+^), showing that increased apoptosis in the naïve T cell population is associated with depletion of naïve T cells (total n = 37, *p*<0.0001, R^2^ = 0.5373). D. Quantification of FRCs (the percent area staining positive for desmin in T cell zone) and the number of apoptotic naïve T cells (TUNEL^+^CD45RA^+^CD3^+^), showing that the depletion of FRCs is associated with increased apoptosis in naïve T cell populations (total n = 37, *p*<0.0001, R^2^ = 0.5843). E. Quantitative image analysis of FRCs and the number of naïve T cells within LTs, showing that the loss of naïve T cells is associated with loss of FRCs (total n = 37, *p*<0.0001, R^2^ = 0.5166). Values are the mean of measurement ± s.d.

These ex vivo co-culture results support the concept that naïve T cells need to contact FRCs in order to gain access to IL-7 to maintain their survival. Therefore, the loss of FRCs as well as the loss of the contact between naïve T cells and FRCs together in vivo would be expected to increase apoptosis and thereby deplete naïve T cells. We tested this hypothesis in LTs from HIV-1 infected patients and found that the stage-dependent increases in naïve T cell apoptosis (Figure 3A) were associated with depletion of both naïve CD4^+^ and CD8^+^ T cells (Figure 3B–C), and that stage-dependent decreases in the FRC network (Figure 1D) were associated both with apoptosis and naïve T cell depletion (Figure 3D–E).

### Restoration of LT structure and increases in naïve T cells depend on the timing of initiation of HAART

Because the LT damage-mediated naïve T cell depletion mechanism now documented in both HIV-1 infection and SIV infection [24] is cumulative and progressive, the later stage of infection, the greater the damage to LT structure. Thus, if treatment does not restore the LT structure that supports survival of naïve CD4^+^ T cells, the LT damage mediated naïve T cell depletion could adversely affect immune reconstitution, even with suppression of viral replication and immune activation by HAART. Conversely, the lesser extent of LT damage in early infection could improve immune reconstitution with HAART, if initiating treatment were to restore LT structure and improve naïve T cell survival.

**Figure 4:**
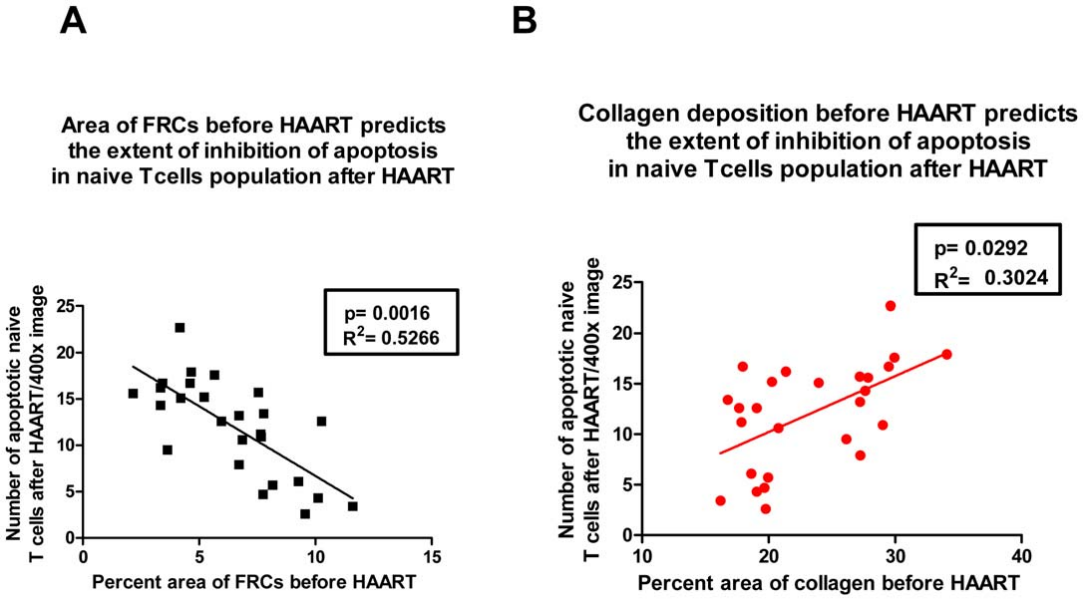
The extent of LT destruction before HAART predicts the extent of restoration of naïve T cells after HAART. A. The area that FRCs occupy before HAART is negatively associated with the number of apoptotic naïve T cells after 6 months of HAART. B. The collagen area before HAART is positively associated with the number of apoptotic naïve T cells after 6 months of HAART.

To test these predictions, we examined the effects of HAART initiated in the acute/early, pre-symptomatic and AIDS stages of infection on LT structure, naïve T cell apoptosis and restoration of naïve CD4^+^ T cell populations. Because loss of the FRC network and fibrosis are less in the acute/early stage than at later stages of HIV-1 infection (Figure 1), we would expect that the preservation of LT structure in acute/early HIV-1 infection would result in decreased apoptosis and greater increases in naïve T cells if HAART is initiated at this stage. We indeed found that the loss of the FRC network and collagen deposition prior to initiating HAART are associated with significantly increased naïve T cell apoptosis after 6 months of HAART (*p* = 0.0016 and *p* = 0.0292 respectively) (Figure 4A–B). Furthermore, the level of naïve T cell apoptosis both before and after treatment is significantly associated with fewer naïve T cells in LTs (*p* = 0.0012). Taken together, these data suggest the extent of LT damage is associated with the extent of inhibition of naïve T cell apoptosis after HAART. We note that this now documents in LTs the previously reported predictive relationship between collagen in LTs and naïve CD4^+^ T cell increases in peripheral blood [27].

**Figure 5:**
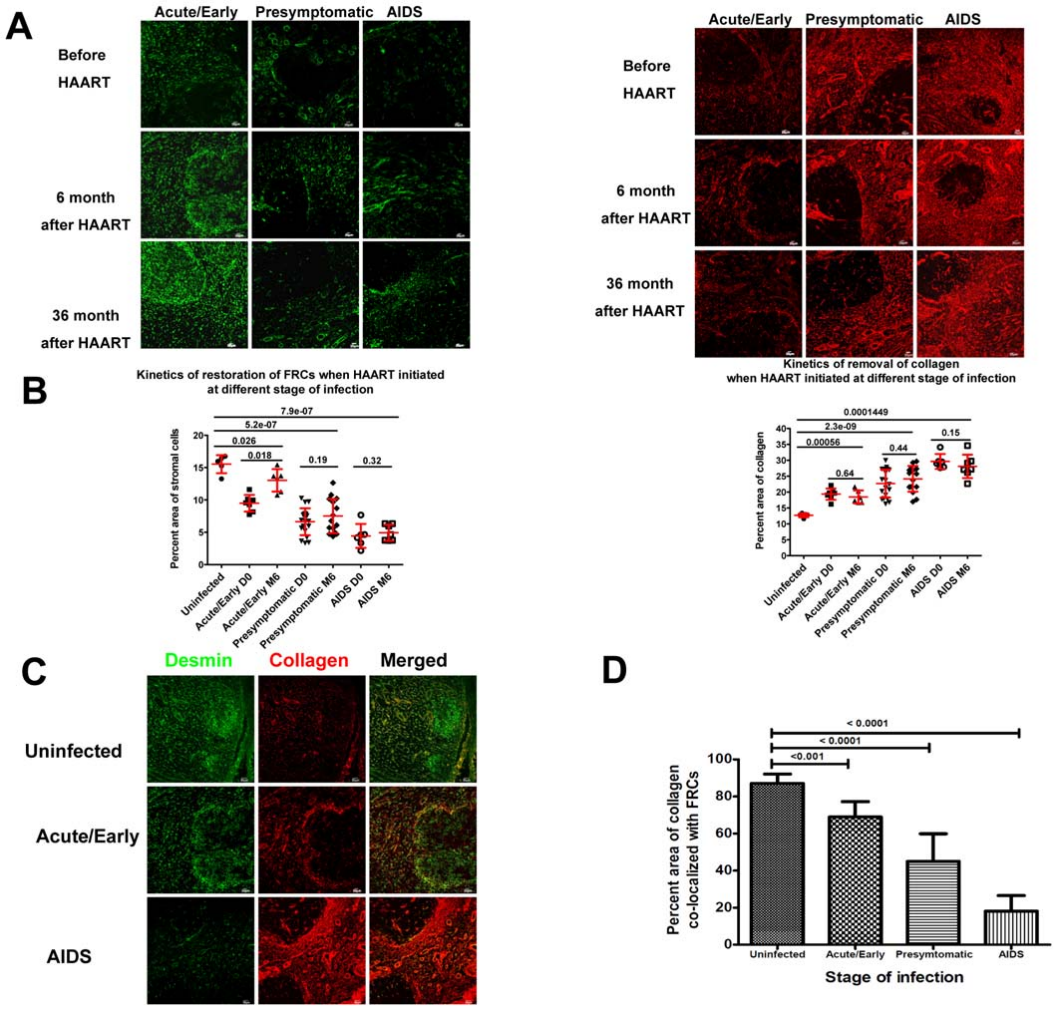
Restoration of LT structure is slow and incomplete after HAART and is associated with the timing of initiation of HAART. A. Representative confocal images of immunofluorescent staining for desmin (green) and collagen (red), showing the different extent of restoration of stromal cell network and collagen normalization after 6 months of HAART. Scale bar, 20 µm. B. Quantification of the average area of FRCs and collagen before and after 6 months of HAART in patients receiving the HAART at different stages of infection, showing the different extent of restoration of LTs is associated with the timing of initiation of HAART. Error bars represent the s.d. C. Representative confocal images of immunofluorescent staining for desmin (green) and collagen (red), showing that the different extent of restoration of the FRC network and collagen normalization after 6 months of HAART as represented by the percent area not covered by FRCs. Scale bar, 20 µm. D. Quantification of the percent area covered by FRCs.

We also find that HAART initiated in the acute/early stage infection results in the greatest restoration of FRCs after 6 months of HAART, albeit not to the level in HIV-1 uninfected population (Figure 5A–B). The restoration of LT structure is a slow process as even after 36 months of HAART the area occupied by the FRC network increases but still remains significantly less than in HIV-1 uninfected individuals. Minimal recovery of the FRC network is seen in HIV-1 infected patients starting HAART at chronic stages (Figure 5A). At 6 months, there is no significant effect on removal of collagen deposition in LTs (Figure 5A–B), but there is increased collagen co-localizing with the FRC network, as opposed to collagen deposits outside the network, albeit not to the same levels seen in HIV-1 uninfected individuals where 90 percent of collagen is within the FRC network (Figure 5C-D). We further found that the new WHO guidelines for initiating HAART at CD4 counts of 350 cells/µl have a sound rationale in preservation of the FRC network, since only when therapy had been initiated at or above 350 cells/µl could we detect significant improvement of FRCs after 6-month HAART (Figure S1).

**Figure 6:**
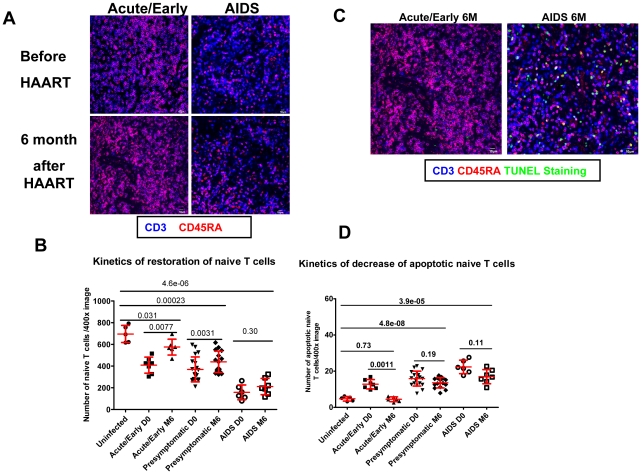
Incomplete LT restoration is associated with high level of apoptosis in naïve T cell populations and incomplete restoration of naïve T cells. A. Confocal images of LN sections from uninfected subjects and patients receiving 6 months of HAART at either acute or AIDS phase of infection, immunofluorescently stained for CD45RA (red) and CD3 (blue), showing the different extent of restoration of naïve T cells. Scale bar, 10 µm. B. Quantification of number of naïve T cells before and after HAART at each stage of infection, showing the different kinetics of restoration of naïve T cells. Error bars represent the s.d. C. Confocal images of LN sections from patients receiving 6 months of HAART at either acute or AIDS phase of infection immunofluorescently stained for CD45RA (red), CD3 (blue) and TUNEL staining of apoptotic cells (green), showing the higher level of apoptosis in naïve T cell populations after 6 months of HAART when HAART was started during chronic phase of infection. Scale bar, 10 µm. D. Quantification of average number of apoptotic naïve T cells before and after HAART at each stage of infection, showing the different kinetics of decrease of apoptotic naïve T cells.

HAART initiated in acute/early stage of infection is also associated with greatest decreases in apoptosis and optimal restoration of naïve T cell populations in LTs (Figure 6). In contrast, naïve T cell numbers in patients who initiated HAART in the AIDS stage of infection did not increase significantly, and apoptosis in naïve T cell populations remained high (Figure 6). These stage-related correlations apply as well to peripheral CD4^+^ T cell counts in patients receiving HAART for 12 months. The increase in peripheral CD4^+^ T cell counts to the level of counts in HIV-1 uninfected individuals depended on initiating HAART during the acute/early stage of infection (Figure S2A). We also found that this stage-dependent restoration of peripheral CD4^+^ T cells can be predicted by the extent of fibrosis before initiation of HAART, again suggesting that fibrosis is one key factor that limits immune reconstitution after long-term HAART (Figure S2B).

**Figure 7:**
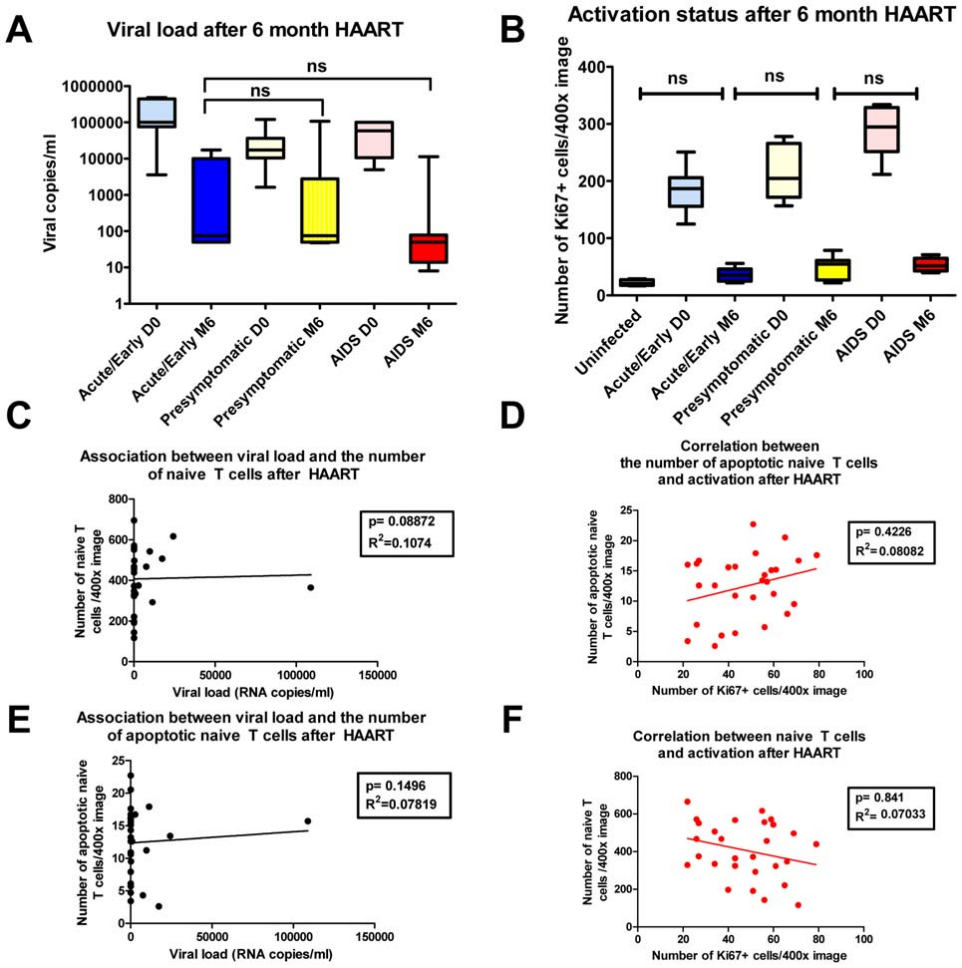
The extent of restoration of naïve T cells after HAART is not associated with viral load or immune activation. A. Viral load before and after HAART in patients receiving HAART at different stages of infection, showing the inhibition of viral replication is similar for patients at different stage of infection. B. Quantification of the number of Ki67^+^ cells before and after HAART in patients receiving HAART at different stages of infection, showing the inhibition of immune activation by HAART is similar for patients at different stage of infection (ns, not significant). C, E. Association between the viral load and number of naïve T cells and association between viral load and the number of apoptotic naïve T cells after HAART are not significant. D, F. Association between the number of Ki67^+^ cells and naïve T cells and association between the number of Ki67^+^ cells and the number of apoptotic naïve T cells after HAART are not significant.

We also assessed the relationships between viral load and the residual Ki67 level to the extent of restoration of naïve T cells when HAART was initiated at different stages. We found that increases in naïve T cells and decreases in apoptosis do not correlate with HAART-mediated suppression of viral replication and immune activation. HAART initiated at all stages of infection can potently and comparably inhibit viral replication and associated chronic immune activation (Figure 7A and B). There is therefore no evidence that these processes are playing important roles in the stage-specific effects of time of initiation on apoptosis and recovery of naïve T cells within LTs. We in fact found no significant association between viral load and the number of naïve T cells or the number of apoptotic naïve T cells, nor any association between activation represented by the number of Ki67^+^ cells and the number of naïve T cells or the number of apoptotic naïve T cells after 6 month HAART (Figure 7C–F).

## Discussion

It has generally been thought that viral and immune-cell mediated killing of CD4^+^ T cells and AICD accompanying chronic immune activation are respectively the major direct and indirect mechanisms of CD4^+^ depletion in HIV-1 infection, and that the slow and incomplete restoration of naïve CD4^+^ T cells is a consequence of the restricted capacity of the adult thymus to re-supply naive T cells [1]. Here we describe a novel mechanism that depletes CD4^+^ T cells, particularly naïve CD4^+^ T cells before HAART and determines the pace and extent of naïve CD4^+^ T cell restoration after HAART.

Naïve T cells depend on IL-7 produced and presented by the FRC network for survival. Our IL-7 staining is consistent with a large literature on the FRC network as the site and source of most of the IL-7 [22], [24], [28]–[29], although different from one report on human IL-7 staining in LTs [30], probably due to different reagents and methodologies used. As HIV-1 infection advances, collagen deposition increases and the FRC network is destroyed, which decreases the amount of IL-7 available to support T cell survival. As a consequence, increased apoptosis depletes naïve T cells prior to HAART in proportion to the progressive destruction of LT structure from early to later stages of HIV-1 infection. Because of the progressive and cumulative nature of this pathological process, apoptosis of naïve T cells continues at elevated levels after HAART has been initiated, even though HAART can potently suppress viral replication and at least partially normalize immune activation. The elevated apoptosis level in naïve T cell populations is in proportion to pre-existing damage to LT structure, which is greater in the chronic stages of infection. Thus, predictably, early treatment results in better preservation and restoration of LT structure, which leads to improved survival of naïve T cells and greater increases in naïve T cell numbers. While the limited capacity of the thymus in the adult to supply naïve T cells will certainly limit the pace and extent of reconstitution, the LT structure to which thymic emigrants home thus also determines their subsequent survival. By analogy to earlier tap-and-drain models [31], restoration of naïve T cells will be dependent not only on thymic output and from homeostatic proliferation of naïve T cell populations in LT, but also on the drain of overall apoptosis in this population. In such a model, the elevated level of apoptosis in naïve T cells in secondary LTs limits both the extent and pace of immune reconstitution.

As the diversity of the naïve T cell repertoire is critical to defend against new infections and malignancies, the loss and slow restoration of the naïve T cell population creates “holes” in the T cell repertoire and therefore impairs host defenses even after HIV-1 replication has largely been suppressed [5], [8]–[9]. Thus, therapeutic approaches to prevent or moderate damage to the LT niche and restore a functional FRC network could be particularly beneficial in increasing and preserving naïve T cell populations after HAART. The most straightforward way to do this is through earlier treatment. Our findings also suggest the potential clinical benefit of complementing IL-7 treatment during HIV-1 infection in the restoration of naïve T cell population. Indeed, studies have shown that complementing HAART with IL-7 during both SIV and HIV infection significantly increases the circulating naïve CD4^+^ T cell number [32]–[34]. Furthermore, it has been shown that IL-7 treatment could normalize the extent of apoptosis in CD4^+^ and CD8^+^ T cells from HIV-1-infected individuals via up-regulation of Bcl-2 levels [35]–[36]. These data consistently suggest that insufficient IL-7 is a key contributor in the impaired T cell homeostasis in SIV/HIV infection and limits the reconstitution of T cells.

However, the immediate decline of the absolute numbers of both naïve CD4^+^ and CD8^+^ T cells after termination of IL-7 therapy [32]–[34] suggests that complementing IL-7 only provides transient survival benefit for naïve CD4 and CD8 T cells and strongly argues for the development of therapeutic interventions to provide long-term survival benefit for naïve T cells through preservation or restoration of the FRC network where naïve cells can access IL-7. Our findings here clearly suggest that collagen deposition and the consequential loss of FRCs as the major source of IL-7 play critical roles in compromising homeostasis of naïve T cells. Therefore, the restoration of the lymphoid tissue niche could potentially provide long-term survival benefits for naïve T cells. Moreover, the development of adjunctive anti-fibrosis treatment such as pirfenidone and losartan [24], [37]–[42] might additionally avert or revert the consequences of damage to the LT niche and improve immune reconstitution.

## Materials and Methods

### Ethics statement

This human study was conducted according to the principles expressed in the Declaration of Helsinki. The study was approved by the Institutional Review Board of the University of Minnesota. All patients provided written informed consent for the collection of samples and subsequent analysis.

### LN biopsy specimens

Inguinal LN (LN) biopsies from HIV negative individuals and HIV-1-infected individuals at different clinical stages (7 at acute/early stage, 18 at presymptomatic stage and 8 at AIDS stage. Table 1) were obtained for this University of Minnesota Institutional Review Board-approved study. Viral load measurements were obtained the same day as biopsies. Each LN biopsy was immediately placed in fixative (4% neutral buffered paraformaldehyde or Streck's tissue fixative) and paraffin embedded.

**Table 1:** Demographic characteristics and clinical information of subjects.

Patient	DiseaseStage	Time after HAART	Race	Gender	Age	Peripheral BloodCD4^+^ T Cell Count (Cells/µl)	Plasma HIV-1RNA Levels(Copies/ml)	Opportunistic Infections (n = none reported)
1292	Uninfected	N/A	Caucasian	Male	28	925	Undetectable	N/A
1476	Uninfected	N/A	Caucasian	Female	28	704	Undetectable	N/A
1472	Uninfected	N/A	Caucasian	Female	52	837	Undetectable	N/A
1425	Uninfected	N/A	Caucasian	Male	43	1351	Undetectable	N/A
1442	Uninfected	N/A	Caucasian	Female	45	1124	Undetectable	N/A
1430	Acute	D0	Caucasian	Male	26	683	3610	n
1430	Acute	M6				702	17400	
1458	Acute	D0	Caucasian	Male	51	400	439000	n
1458	Acute	M6				671	<50	
1329	Acute	D0	Caucasian	Male	59	370	484694	n
1329	Acute	M6				871	<50	
1329	Acute	M36				789	<50	
1469	Acute	D0	Caucasian	Male	44	180	>100,000	n
1469	Acute	M6				321	7547	
1449	Acute	D0	Caucasian	Male	30	333	>100000	n
1435	Acute	D0	Caucasian	Male	42	410	>100,000	Unknown
1435	Acute	M6				663	<50	
1391	Acute	D0	Black or African American	Male	37	414	24718	n
1391	Acute	M6				765	<50	
1437	AIDS	D0	Caucasian	Male	47	214	656	n
1437	AIDS	M6				235	<50	
1438	AIDS	D0	Caucasian		49	147	4960	n
1438	AIDS	M6				151	<50	
1438	AIDS	M36				216	<50	
1406	AIDS	D0	Black or African American	Male	45	188	10684	n
1406	AIDS	M6				209	11438	
1413	AIDS	D0	Black or African American	Male	50	42	59401	Unknown
1413	AIDS	M6				121	8	
1462	AIDS	M6				143	<50	n
1327	AIDS	D0	Black or African American	Female	40	112	12046	n
1327	AIDS	M6				180	14	
1419	AIDS	D0	Caucasian	Male	37	157	61432	n
1419	AIDS	M6				320	79	
1463	Pre	D0	Black or African American	Male	23	259	27200	n
1463	Pre	M6				599	<50	
1447	Pre	D0	Caucasian	Male	37	640	12100	Unknown
1447	Pre	M6				776	24300	
1468	Pre	D0	Caucasian	Male	30	875	2150	n
1335	Pre	D0	Caucasian	Male	32	400	15284	n
1335	Pre	M36				458	<50	
1428	Pre	D0	Caucasian	Male	30	363	38600	Unknown
1428	Pre	M6				379	<50	
1479	Pre	D0	Caucasian	Male	42	273	1650	n
1479	Pre	M6				479	<75	
1464	Pre	D0	Caucasian	Male	34	202	122000	n
1464	Pre	M6				450	72	
1669	Pre	D0	Caucasian	Male	23	434	6506	n
1669	Pre	M6				524	85	
1293	Pre	D0	Caucasian	Male	36	905	14225	Unknown
1293	Pre	M6				1842	<50	
1293	Pre	M36				2251	<50	
1408	Pre	D0	Caucasian	Male	39	685	20014	n
1408	Pre	M6				592	9815	
1680	Pre	M6	Caucasian	Male	38	539	<48	n
1448	Pre	D0	Black or African American	Male	51	543	10000	Unknown
1448	Pre	M6				335	2790	
1727	Pre	D0	Caucasian	Male	39	336	17600	n
1436	Pre	D0	Caucasian	Male	63	248	46400	n
1436	Pre	M6				297	893	
1407	Pre	D0	Caucasian	Male	35	372	31922	n
1407	Pre	M6				353	108996	
1679	Pre	D0	Caucasian	Male	36	620	17388	n
1679	Pre	M6				721	<48	
1317	Pre	D0	Caucasian	Male	31	399	120469	n
1317	Pre	M6				779	303	
1766	Pre	D0	Black or African American	Male	36	389	6810	n
1455	Pre	D0	Black or African American	Male	23	209	19400	n
1455	Pre	M6				324	<50	
1459	Pre	D0	Caucasian	Male	36	286	>100000	n

Note: Definition of classification of HIV infection stage: Acute/Early stage: patients are RNA^+^ and antibody negative or have serologic proof of infection within the previous 4 months. AIDS stage: patients whose CD4 count is <200cells/µL. Presymptomatic stage: patients between acute/early stage and AIDS stage.

### Immunofluorescence staining and Quantitative Image Analysis (QIA)

All staining procedures were performed as previously described [24], [43] using 5–30 µm tissue sections mounted on glass slides. Tissues were deparaffinized and rehydrated in deionized water. Heat-induced epitope retrieval was performed using a high-pressure cooker (125°C) in either DIVA Decloaker or EDTA Decloaker (Biocare Medical), followed by cooling to room temperature. Tissues for collagen type I staining required pre-treatment with 20 µg/ml proteinase K (Roche Diagnostics) in proteinase K buffer (0.2 M Tris, pH 7.4, 20 mM CaCl_2_) for 15–20 min at room temperature. Tissue sections were then blocked with SNIPER Blocking Reagent (Biocare Medical) for 30 min at room temperature. Primary antibodies were diluted in TNB (0.1M Tris-HCl, pH 7.5; 0.15 M NaCl; 0.05% Tween 20 with Dupont blocking buffer) and incubated overnight at 4°C. After the primary antibody incubation, sections were washed with phosphate buffered saline (PBS) and then incubated with fluorochrome-conjugated secondary antibodies in TNB for 2 hr at room temperature. Finally, sections were washed with PBS, nuclei were counterstained blue with DAPI, and mounted using Aqua Poly/Mount (Polysciences Inc.). Immunofluorescent micrographs were taken using an Olympus BX61 Fluoview confocal microscope with the following objectives: x20 (0.75 NA), x40 (0.75 NA), and x60 (1.42 NA); images were acquired and mean fluorescence intensities were analyzed using Olympus Fluoview software (version 1.7a).

Isotype-matched negative control antibodies in all instances yielded negative staining results (see Table 2, which lists the primary antibodies and antigen retrieval methodologies).

**Table 2:** List of primary antibodies and antigen retrieval methodologies.

Antibody	Clone/Manufacturer & Catalog #	Antigen-retrieval Pretreatment	Antibody Dilution	Species
Desmin	D33/Lab Vision & # MS-376-S1	Diva Decloaker; High pressurecooker for 30 seconds at 125°C.	1/200	Mouse
Desmin	Polyclonal/Lab Vision & # RB-9014-P1	Diva Decloaker; High pressurecooker for 30 seconds at 125°C.	1/200	Rabbit
CD3	MCA147/AbD Serotec & # MCA1477	Diva Decloaker; High pressurecooker for 30 seconds at 125°C.	1/200	Rat
CD3	SP7/Thermo Scientific & # RM-9107-S1	Diva Decloaker; High pressurecooker for 30 seconds at 125°C.	1/100	Rabbit
IL-7	7417/R & D Systems & # MAB207	Diva Decloaker; High pressurecooker for 30 seconds at 125°C.Proteinase K treatment for 15 min	1/100	Mouse
CD45RA	4KB5/Dako & # M0754	Diva Decloaker; High pressurecooker for 30 seconds at 125°C.	1/100	Mouse
ActivatedCaspase-3	8G10/Cell Signaling Tech. & # 9665	1mm EDTA (ph 8); High pressurecooker for 30 seconds at 125°C.	1/100	Rabbit
Collagen 1	COL-1/Sigma & # C2456	Diva Decloaker; High pressure cookerfor 30 seconds at 125°C. Protease K (10 µg/ml).	1/100	Mouse
Collagen I	Polyclonal/Abcam & # ab292	Diva Decloaker; High pressure cooker for30 seconds at 125°C. Protease K (10 µg/ml).	1/200	Rabbit
CD4	Polyclonal/R & D Systems & # AF-379-NA	Diva Decloaker; High pressure cooker for30 seconds at 125°C.	1/100	Goat
CD4	1F6/Novacastra & # NCL-CD4–1F6	Diva Decloaker; High pressure cooker for30 seconds at 125°C.	1/100	Mouse
CD8	SP16/Neomarkers & # RM-9116-s	Diva Decloaker; High pressure cooker for30 seconds at 125°C.	1/100	Rabbit
Ki-67	SP6/Neomarkers & # RM-9106-S1	Diva Decloaker; High pressure cooker for30 seconds at 125°C.	1/200	Rabbit
IgG IsotypeControls	Dako, Jackson ImmunoResearch	Diva Decloaker; High pressure cooker for30 seconds at 125°C. Protease K (10 µg/ml).	1/50-1/200	Mouse, Rabbit, Rat, Goat

A list of ID numbers for genes and proteins used in the paper: Desmin: 1674, CD3: 916, Interleukin-7: 3574, CD45RA: 151460, Caspase3: 600636, Collagen Type I: 120150, CD4: 186940, CD8: 925, Ki-67: 176741.

Quantitative image analysis (QIA) was performed using 10–20 randomly acquired, high-powered images (X200 or X400 magnification) by either manually counting the cells in each image or by determining the percentage of LT area occupied by positive fluorescence signal using an automated action program in Adobe Photoshop CS with tools from Reindeer Graphics.

### *Ex vivo* culture system

The experimental protocols used here for human tissue samples had full IRB approval (Institutional Review Board: Human Subjects Committee, Research Subjects' Protection Program, University of Minnesota) and informed written consent was obtained from individual patients, or the legal guardians of minors, for the use of tissue in research applications prior to the initiation of surgery. Fresh human palatine tonsil tissues were obtained from routine tonsillectomies and processed within 1–2 h of completion of surgery. Viable tonsil lymphocyte suspensions were prepared by forcing cut tissue pieces through a metal sieve and collecting the released single cell suspension in complete RPMI medium (10% heat inactivated fetal calf serum, 1x l-glutamine, penicillin, and streptomycin solution; Invitrogen). The cells were washed and immediately cryopreserved. By culturing the stroma left on the metal sieve in complete RPMI medium, adherent proliferating fibroblasts were first visible after 2–5 days in culture, and confluent monolayers developed after 10–25 days. These primary stromal populations were readily released with trypsin, and the cells were further expanded and passaged using routine procedures for adherent cells. Some stromal cells were fixed in Streck's tissue fixative at one day prior to co-culture for analysis of intracellular desmin and IL-7 expression. For live stromal cells staining and imaging, stromal cells were directly incubated with antibody against IL-7 at 4°C without heat antigen retrieval and subject to secondary fluorochrome-conjugated-antibody staining. For co-culture of lymphocytes and stromal cells, 2×10^5^ lymphocytes isolated from human tonsil were cultured in chamber slides without stromal cells, with autologous stromal cells (2×10^4^cells/well), with stromal cells and IL-7 blocking antibody or with stromal cells but separated by transwells for 2 to 3 days. After co-culture, the slides were fixed in Streck's tissue fixative and stained for activated caspase3, CD45RA and CD3 to quantify the number of apoptotic naïve T cells by QIA as described above.

### Statistical analysis

To test for differences in FRCs and collagen across all stages a 1-way ANOVA was used and post-hoc comparisons were made with Welch's modified 2 sample *t*-tests with a Bonferroni correction (hence *p*-values are reported for differences between stages). A similar analysis was used to test for differences from the data that arose from the *ex vivo* culture system.

To test for associations between FRCs and apoptotic naïve cell counts mixed models were used with FRCs as the explanatory variable in addition to clinical stage of infection (since it is associated with both FRCs and apoptotic naïve cell counts). Random effects were included in these models since all of the data (i.e. all time points) were used to fit these models and random effects provide a simple way to incorporate correlation among measurements from the same subject into the model. Continuous variables were log transformed prior to fitting the model and restricted maximum likelihood was used to obtain parameter estimates. The same approach was used to test for an association between apoptotic naïve cell counts and naïve counts. A similar approach was used to test for an association between both Ki67 levels and viral load and apoptotic naïve cell counts and naïve counts except that FRCs and collagen were included in the model in addition to clinical stage of infection.

To test if baseline FRCs or collagen are predictive of apoptotic naïve cell counts and naïve counts at 6 months post initiation of HAART, linear regression models were used that included the baseline value of the variable we were trying to predict at 6 months since such baseline values are potentially related to the value of the variable at 6 months and the baseline levels of FRCs or collagen. All continuous variables were first log transformed and standard model diagnostics were conducted.

One sample *t*-tests were used to test for changes over the first 6 months of therapy for each stage. Spearman's rank correlation was used to test for associations that were potentially nonlinear (but monotone).

## Supporting Information

Figure S1 **Significant restoration of FRCs is associated with early initiation of HAART.** Bar plot shows that the increase of FRCs is significant when HAART is initiated when peripheral CD4^+^ T cells is above 350 cells/µl. In contrast to that, the increase of FRCs is insignificant when HAART is started when peripheral CD4^+^ T cells is below 350 cells/µl (*, *p<*0.05; ns, not significant).
(TIF)

Figure S2 **Incomplete restoration of peripheral CD4 count after 12 month HAART is associated with initiation of HAART during chronic stage of infection.** A. Plot shows that the level of peripheral CD4 count is not significantly different from that in uninfected subjects when HAART is initiated during acute/early stage of infection after 12 month HAART. In contrast to that, when HAART is started during chronic stage of infection, the level of peripheral CD4 count is still significantly lower than that in uninfected subjects after 12 month HAART (*, *p<*0.05; **, *p*<0.01; ***, *p*<0.0001; ns, not significant). B. The percent area of collagen before HAART is negatively associated with the number of peripheral CD4 count after 12 months of HAART.
(TIF)
