# Supplementary figures and images for: Lymphoid Tissue Damage in HIV-1 Infection Depletes Naïve T Cells and Limits T Cell Reconstitution after Antiretroviral Therapy
Source: PLoS Pathog. 2012 Jan 5;8(1):e1002437. doi: 10.1371/journal.ppat.1002437 (PMC3252371; doi:10.1371/journal.ppat.1002437)

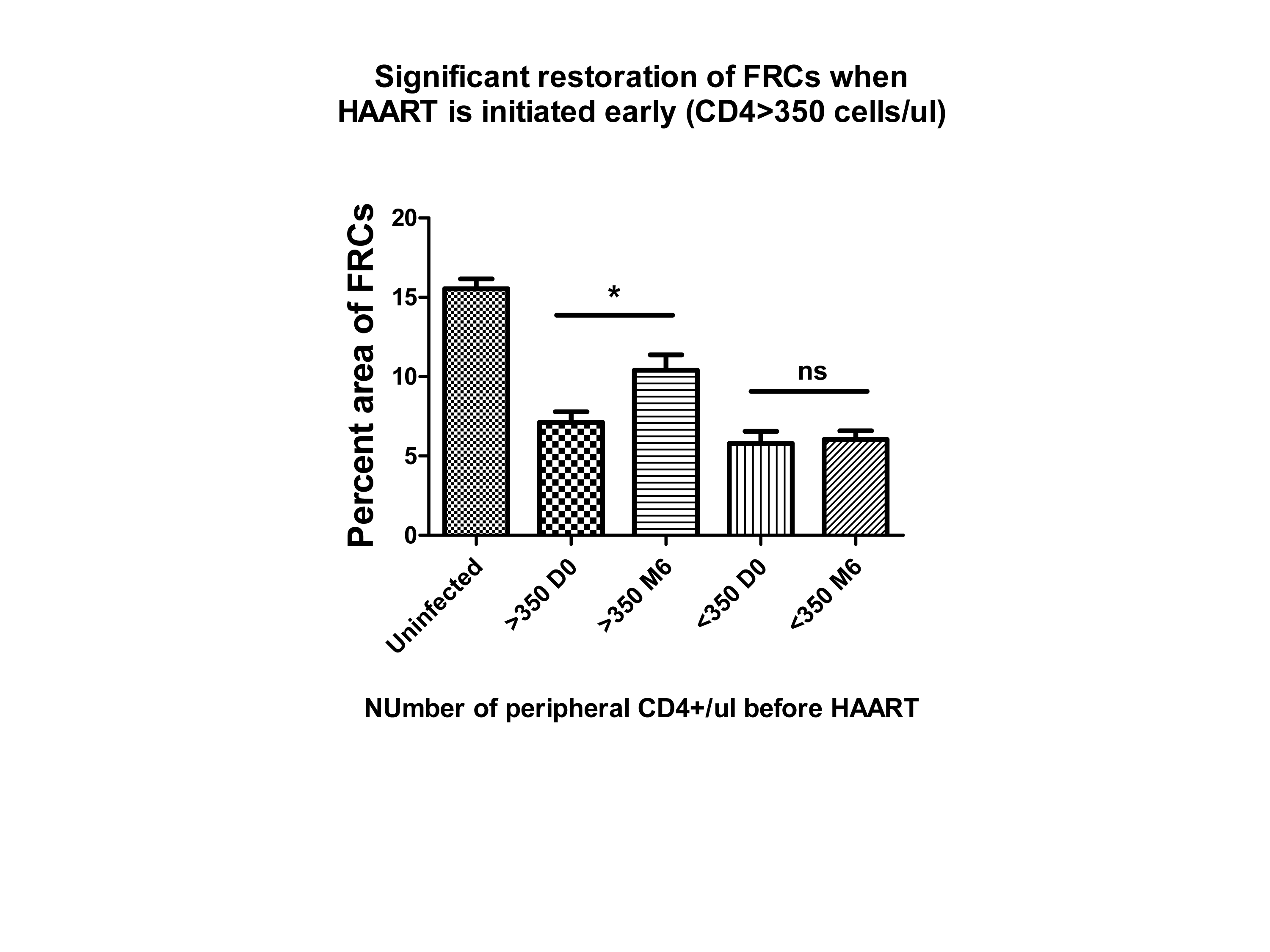

Supplement: Figure S1 — Significant restoration of FRCs is associated with early initiation of HAART. Bar plot shows that the increase of FRCs is significant when HAART is initiated when peripheral CD4+ T cells is above 350 cells/µl. In contrast to that, the increase of FRCs is insignificant when HAART is started when peripheral CD4+ T cells is below 350 cells/µl (*, p<0.05; ns, not significant). (TIF) [file ppat.1002437.s001.tif]

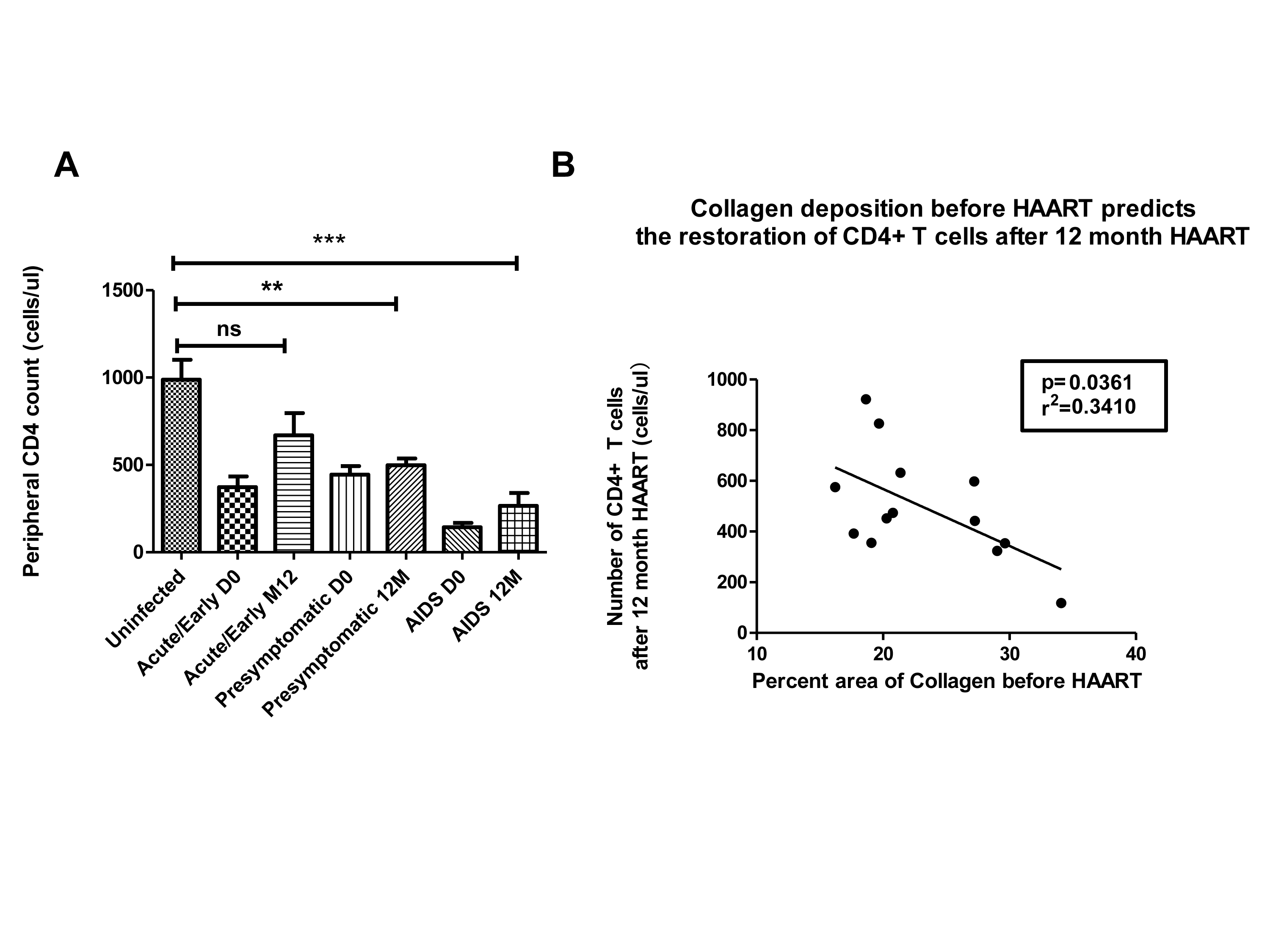

Supplement: Figure S2 — Incomplete restoration of peripheral CD4 count after 12 month HAART is associated with initiation of HAART during chronic stage of infection. A. Plot shows that the level of peripheral CD4 count is not significantly different from that in uninfected subjects when HAART is initiated during acute/early stage of infection after 12 month HAART. In contrast to that, when HAART is started during chronic stage of infection, the level of peripheral CD4 count is still significantly lower than that in uninfected subjects after 12 month HAART (*, p<0.05; **, p<0.01; ***, p<0.0001; ns, not significant). B. The percent area of collagen before HAART is negatively associated with the number of peripheral CD4 count after 12 months of HAART. (TIF) [file ppat.1002437.s002.tif]
